# Measuring Airway Surface Liquid Depth in *Ex Vivo* Mouse Airways by X-Ray Imaging for the Assessment of Cystic Fibrosis Airway Therapies

**DOI:** 10.1371/journal.pone.0055822

**Published:** 2013-01-30

**Authors:** Kaye S. Morgan, Martin Donnelley, David M. Paganin, Andreas Fouras, Naoto Yagi, Yoshio Suzuki, Akihisa Takeuchi, Kentaro Uesugi, Richard C. Boucher, David W. Parsons, Karen K. W. Siu

**Affiliations:** 1 School of Physics, Monash University, Melbourne, Victoria, Australia; 2 Women's and Children's Hospital, Adelaide, South Australia, Australia; 3 Department of Paediatrics and Reproductive Health, University of Adelaide, Adelaide, South Australia, Australia; 4 Centre for Stem Cell Research, University of Adelaide, Adelaide, South Australia, Australia; 5 Department of Mechanical and Aerospace Engineering, Monash University, Melbourne, Victoria, Australia; 6 SPring-8/Japan Synchrotron Radiation Research Institute, Kouto, Hyogo, Japan; 7 Cystic Fibrosis Research and Treatment Center, University of North Carolina, Chapel Hill, North Carolina, United States of America; 8 Monash Biomedical Imaging, Monash University, Melbourne, Victoria, Australia; 9 Australian Synchrotron, Melbourne, Victoria, Australia; University of Tübingen, Germany

## Abstract

In the airways of those with cystic fibrosis (CF), the leading pathophysiological hypothesis is that an ion channel defect results in a relative decrease in airway surface liquid (ASL) volume, producing thick and sticky mucus that facilitates the establishment and progression of early fatal lung disease. This hypothesis predicts that any successful CF airway treatment for this fundamental channel defect should increase the ASL volume, but up until now there has been no method of measuring this volume that would be compatible with *in vivo* monitoring. In order to accurately monitor the volume of the ASL, we have developed a new x-ray phase contrast imaging method that utilizes a highly attenuating reference grid. In this study we used this imaging method to examine the effect of a current clinical CF treatment, aerosolized hypertonic saline, on ASL depth in *ex vivo* normal mouse tracheas, as the first step towards non-invasive *in vivo* ASL imaging. The *ex vivo* tracheas were treated with hypertonic saline, isotonic saline or no treatment using a nebuliser integrated within a small animal ventilator circuit. Those tracheas exposed to hypertonic saline showed a transient increase in the ASL depth, which continued for nine minutes post-treatment, before returning to baseline by twelve minutes. These findings are consistent with existing measurements on epithelial cell cultures, and therefore suggest promise for the future development of *in vivo* testing of treatments. Our grid-based imaging technique measures the ASL depth with micron resolution, and can directly observe the effect of treatments expected to increase ASL depth, prior to any changes in overall lung health. The ability to non-invasively observe micron changes in the airway surface, particularly if achieved in an *in vivo* setting, may have potential in pre-clinical research designed to bring new treatments for CF and other airway diseases to clinical trials.

## Introduction

To maintain normal lung function, it is essential that the airways clear cellular debris or pathogens that deposit during respiration. The surface of the conducting airways are covered with microscopic hairs called cilia, located within the airway surface liquid (ASL), that provide the driving force for mucociliary clearance (MCC). The ASL comprises two layers, the periciliary liquid (PCL) (typically 4–6 µm deep in the nasal airways of normal mice), and an overlying mucus layer [1]. Debris, bacteria and viruses are normally captured by the mucus layer and transported along the airway surface away from the lungs via cilia action until the material is directed into the oesophagus and swallowed. In the case of larger airway debris or mucus materials, MCC can be supplemented by cough mechanisms [2].

In cystic fibrosis (CF) a defect in the cystic fibrosis transmembrane conductance regulator (CFTR) gene produces a chloride-ion channel defect in epithelial cells [1]. In the conducting airways, this defect results in a reduction in the ASL volume [3], [4] and produces a relative dehydration of the airway surface such that MCC is compromised. The thickened and sticky mucus cannot be sufficiently cleared by MCC, resulting in retention of both mucus and inhaled pathogens. These pathogens contribute to the persistent infection that underlies the progressive lung disease and decreased respiratory function that is typical of CF [2]. Although CF impairs the function of a range of other organ systems, it is the chronic infectious airway and lung diseases that is the primary cause of a decreasing quality of life and an early death in most CF patients.

Therapies that focus on the correction of abnormal epithelial cell ion transport or the CFTR defect itself are attractive as they have the potential to alter lung disease progression and hence patient prognosis [5]. Currently, the inhalation of hypertonic saline or mannitol is used in the CF clinic to rehydrate the ASL by altering the osmotic gradient across the airway surface, and drawing water onto the airway cell surface [6], [7], [8]. Airway gene therapies are designed to insert a correctly functioning copy of the CFTR gene into the epithelial cells to correct the source of the ion channel defect and, consequently, so restore ASL volume [9]. The newest treatment for CF airway disease is the FDA approved drug Ivacaftor, a chloride channel potentiator specifically for the G551D CF mutation [10]. Regardless of their mechanism of action, treatments that increase the airway surface liquid volume and improve MCC, should improve quality of life and extend the lifespan in people with CF [1].

Despite the opportunity afforded by these ASL modifying treatments to alter disease progression, there are no rapid or direct methods that non-invasively assess treatment effectiveness at the primary site of action – the airway surface – where ASL volumes are altered. In humans, CF health is currently estimated by measuring changes in forced expiratory volume (FEV), and more recently by studying changes in lung structural disease, as assessed by CT [11]. These methods can indicate whether a treatment has altered progression of established lung disease, but measurements must be taken over several months. Similarly, disease must be largely established to be detectable using these methods and hence, they cannot detect the initiation of CF lung disease. Although ASL volume measures have previously been estimated in airway epithelial cultures [3], [12], [8] and in fragments of trachea [13], by measuring the ASL height these methods are clearly not compatible with *in vivo* monitoring. There exists a continuing demand for a method that can immediately detect the presence of CF airway surface disease, and provide immediate feedback in treatment assessment. Direct measurement of the ASL volume (or depth) before and immediately after treatment would enable early, rapid and iterative treatment with the potential for modifying the disease-establishment process.

We have developed a synchrotron x-ray imaging technique with sufficient resolution and sensitivity to quantify the ASL depth within an intact trachea [14], [15]. The method uses short x-ray exposures to capture images of the living airway, therefore avoiding motion blur, and is amenable to *in vivo* application since penetrating x-rays can image structures deep within the body at very high resolutions. The ability to resolve soft tissue structures using x-rays is realized by a mechanism called phase contrast. Phase contrast x-ray imaging (PCXI) visualises changes in the phase of the x-ray wavefield as well as changes in intensity. Phase variations can be converted into an observable intensity image via a variety of experimental set-ups that include propagation-based imaging [16], [17], analyzer-based imaging [18] and Talbot interferometry with gratings [19], [20]. It is difficult to capture moving live biological activity with these latter two methods of PCXI since they usually require either multiple exposures while moving the optics, or a longer exposure to compensate for loss of intensity in the optics.

Live animal imaging studies typically use propagation-based PCXI, where a distance is introduced between the sample and camera (typically tens of centimeters to a few metres), so that bright-dark intensity fringes are propagated from sharp interfaces between differing materials in the sample onto the detector/camera, creating an image with enhanced edge visibility [16], [17], [21], [22]. Edges running in any direction are captured within a single short exposure. However, propagation-based PCXI has relatively limited contrast sensitivity (compared to other phase contrast imaging methods) and cannot easily detect smoother edges or subtle changes in thickness. In particular, we have previously reported that propagation-based PCXI can readily resolve the ASL-to-air interface, but the ASL-to-tissue interface has hitherto not been directly visible since these two materials have very similar densities and composition [14].

To obtain sufficient contrast resolution to image the ASL-to-tissue interface, we have developed a Shack-Hartmann-like [23], [24], [25] PCXI method. This method is sensitive to the transverse gradients (mathematically, the first derivatives) present in the sample, rather than the second derivative, as is the case with propagation-based PCXI [26]. This additional sensitivity is achieved by utilizing a single absorption grid, placed before the sample (the airway), which also allows us to measure phase variations in both directions within a single short exposure [14], [27]. Our method can be illustrated in a visible light example by analogy to the distorted shadow of a fence observed on the bottom of a swimming pool. The sun (analogous to the “x-ray source”) illuminates the fence (the absorption “grid”), and the fence-patterned light passes through the water (here representing the sample), landing on the bottom of the pool (the “camera”). The lens-like effect of the water surface will distort the shadow of the fence when seen on the bottom of the pool. By looking at how the fence pattern has been distorted by the water, an image describing the water surface can be reconstructed. The airway sample has a similar effect on the x-rays as the water does on the visible light - changing the ray path of the light, without significantly decreasing the intensity. In the same way the image of the pool surface can be reconstructed, so can the image of the airway. Note that while there will be low-contrast patterns on the bottom of the pool from the water surface alone (analogous to propagation-based PCXI), the addition of the high-contrast reference fence pattern will allow more subtle surface variations to be detected, resulting in a more sensitive imaging system.

This study utilised our grid-based imaging technique to examine the ASL in an intact, fastly excised mouse trachea, maintained in a warm biological saline bath. The *ex vivo* sample avoids the confounding structures introduced by overlying tissue and skin present, and so *ex vivo* imaging is the first step in measuring the depth of the low-contrast ASL. Using this new imaging approach we demonstrate here that ASL depth changes induced by treatments can be rapidly and directly measured. The ability to track ASL depth changes in *ex vivo* samples provides the basis for non-invasive *in vivo* and long-term repeated imaging.

## Methods

### Ethics Statement

All animal experiments were approved by the Animal Ethics Committee of SPring-8 (proposal 2011A1306) and the Women's and Children's Health Network, Adelaide (AE805-12-2012). All animals were humanely killed before surgery began to minimize suffering.

### Sample preparation

**Figure 1:**
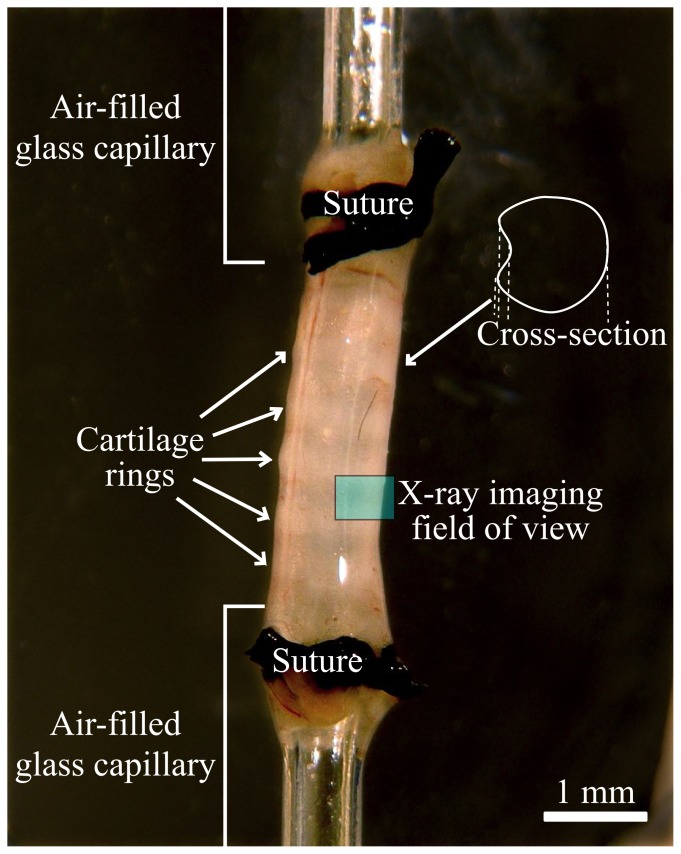
*Ex vivo* trachea mounted vertically between air-filled capillaries and submerged in a warm phosphate buffered saline bath for imaging. The sample is arranged so that the imaged ASL region is the ventral tracheal surface.

Immediately after humane killing by Nembutal overdose, tracheas were removed as a single piece from the carina to the cricoid cartilage from female C57BL/6 mice (total n = 15, ∼19 grams body weight), using standard dissection techniques. Each end of the trachea was sutured onto air-filled glass capillary tubes (0.8 mm outer diameter), as shown in Fig. 1, and extended to mimic the in-animal between-cartilage lengths. After excision, ciliary beat was visible via stereo-microscope through the wall of the trachea, confirming active MCC. The capillaries were connected to a flexiVent ventilator (SCIREQ, Canada), and the arrangement submerged in 37°C phosphate buffered saline (PBS) within a sealed tissue chamber (Living Systems CH-1, USA), modified with the addition of a reduced depth x-ray imaging section (to minimise x-ray absorption for high x-ray flux and short exposures). The tissue-bath was mounted for imaging so that the trachea was positioned vertically (Fig. 1) and the ventral side of the airway was imaged. The ventral side was chosen to avoid multiple edges appearing in the images, as can be present on the dorsal side of the trachea where airway surface projection into the lumen can occur due to the size of the underlying trachealis muscle (see Fig. 1). To mimic in-animal conditions used in our live animal studies, the trachea was ventilated with room air at 80 breaths/minute, a tidal volume of 0.4 ml and a PEEP (positive end expiratory pressure) of ∼3 cm H_2_O.

Each trachea was randomly assigned to receive either no treatment, isotonic saline (0.7%) or hypertonic saline (7%) (with n = 5 for each group).

Treatments were delivered using an Aeroneb Pro nebuliser (Aerogen, Ireland), designed to produce 4–6 µm volume median diameter (VMD) aerosols, with delivery controlled by the flexiVent ventilator. Aerosols were delivered with 50% duty cycle, during inspiration only, for a period of 90 seconds. Images were captured immediately before treatment delivery, then at 3 minute intervals after the initial imaging up to a total of 18 minutes (totaling seven time-points). Ten images were taken at each time-point. A mechanical shutter shielded the trachea from the x-ray beam between image captures to minimise the radiation dose.

### Imaging method

Imaging was performed at the SPring-8 synchrotron in Japan, using the BL20XU beamline in the Biomedical Imaging Centre (245 m from the storage ring). This undulator x-ray beamline provides highly coherent x-rays for detailed phase contrast, high flux for short exposures, and a suitably large area of illumination. The x-ray energy was set at 25 keV, sufficiently high that sample absorption did not lead to unacceptably long exposures and sufficiently low that good phase contrast was achieved.

Images were captured using a pco.4000 (PCO imaging) CCD camera, coupled to a 10 µm thick scintillator (to convert x-rays to visible light) and a 50× microscope objective lens to give an effective pixel size of 0.18×0.18 µm. The CCD provided 4008×2672 pixels, hence a 721×497 µm field of view was available. The 150 ms exposure time was sufficiently short that ventilation induced movement of the airway surface did not create movement blur in the images.

**Figure 2:**
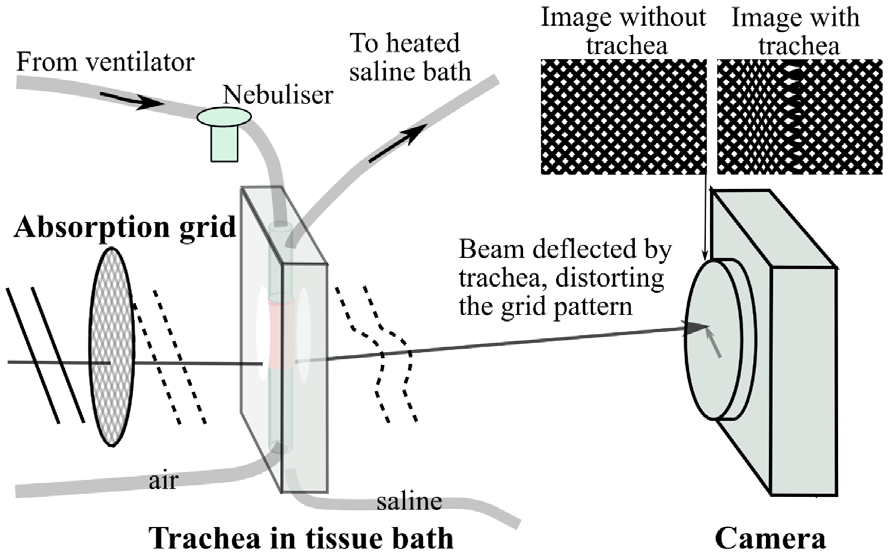
Single-exposure single-grid phase contrast x-ray imaging set-up, for imaging of a live trachea in a specially-built tissue bath.

The simple experimental set-up is shown in Fig. 2, where an x-ray source illuminated a gold absorption grid (25.4 µm period, G1000HS-G3, Gilder Grids, UK), after which the grid-patterned x-rays passed through the mouse trachea and propagated 1 metre to the camera. Phase variations introduced by the mouse trachea distort the intensity grid image that was captured by the camera.

**Figure 3:**
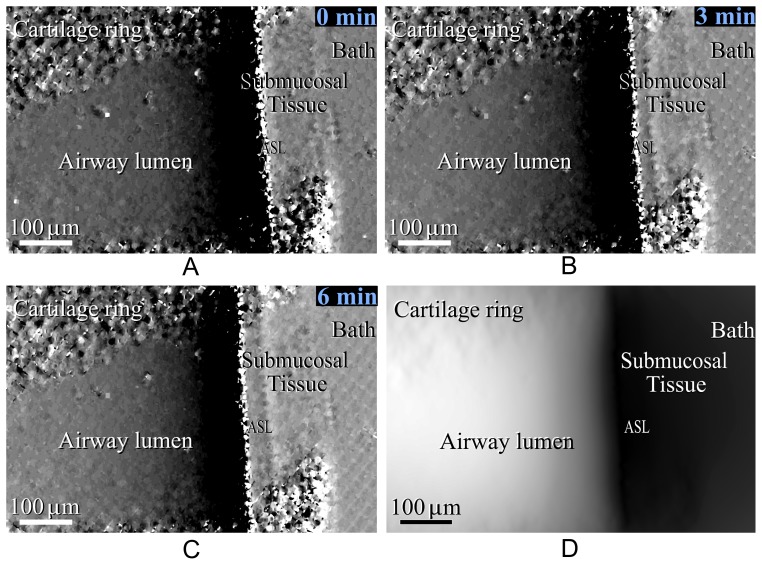
Single-grid based image of the airway surface under hypertonic saline treatment, showing the sample thickness gradient in the *x* direction (the thickness gradient in the *y* direction is not shown as most features run vertically and do not provide *y-*contrast) *a)* before hypertonic saline treatment, *b)* 3 minutes after treatment, *c)* 6 minutes after treatment and *d)* the projected thickness calculated from image *c)*.

The grid was imaged (“Image without trachea” in Fig. 2) before the trachea was introduced and this undistorted reference image was then compared to each image of the grid and trachea (“Image with trachea” in Fig. 2) captured over the time-points before and after treatment. The distortion the sample imparted on each part of the grid image was determined using a cross-correlation technique [14], which revealed how far and in which direction the pattern was shifted at each pixel in the image. An image where the intensity of each pixel is the shift in the horizontal direction at that pixel position will describe the *x* direction gradient of the sample's thickness and density, as seen in Fig. 3*a*. In the case of a vertical trachea, as seen here, the vertical component of the shift (the *y* direction sample gradient) is very small and provides little information, as the sample varies mainly in the *x* direction. The two gradient images can be integrated together to give an image of the total density-thickness [14], which is comparable to a conventional clinical x-ray image (i.e. the intensity is directly related to the thickness and density of the sample), Fig. 3*b*.

Since the sharp diagonal grid lines will intersect with the vertical airway interfaces in the “Image with trachea”, the spatial resolution in the local position of the airway interface at the edge of each grid line will be roughly equivalent to the resolution of the imaging system (∼1 µm). While the same local edge position resolution will not be achieved in the space between grid edges, our previous propagation-based PCXI data has indicated that at these length scales (i.e. the vertical distance between grid edges – a maximum of ∼10 µm in this case) the interfaces will vary smoothly (particularly the liquid interface, which has surface tension), leading to an accurate measure down the length of trachea in the field of view. This is in fact like taking an interpolated average of measurements every <10 µm down the trachea length.

### Analysis

ASL depth was measured by manually tracing both the airway/ASL interface and the ASL/tissue interface in the sample gradient image (Fig. 3*a*), tracking along between the dark/light bands of each interface. The manual placement of these lines had an acceptably high intraclass correlation of 0.992 (95% confidence limits of 0.987–0.997) for independent placement by 5 different scorers on 9 images. The mean distance was then computationally measured between these two interface traces (airway/ASL and ASL/tissue) for every row of pixels down the length of airway present between cartilage rings. This measurement was repeated at each of the 7 timepoints, for each of the 15 mouse tracheas (n = 5 for three “treatments”), blinded to treatment.

Statistical analyses were performed using GraphPad Prism 5. The ASL data was square root transformed to ensure normality assumptions were met. Statistical significance was set at p = 0.05 and power = 0.8 and measures were analysed by two way repeated measures ANOVA with Bonferroni multiple comparisons. The depth measures are presented as scatter plots with means.

## Results

A typical airway surface image obtained using the single-grid PCXI method is shown in Fig. 3*a, b and c*. The interface between the air-filled trachea and the ASL is clearly visible, with a steep phase gradient (i.e. seen as increasingly dark/black) ending at the ASL on the edge of the airway lumen. A less distinct line is seen further to the right; this is taken as the ASL/tissue interface. This latter interface is not visible using a standard propagation-based PCXI set-up. The presence of both the ASL interfaces in our images means that ASL depth measurements can be made. Note the speckled regions at the top and bottom of the image are from the tracheal cartilage rings. It can be seen that the ASL depth increases after hypertonic saline is delivered (immediately after Fig. 3*a*, 0 min) with the two interfaces moving further apart in Fig. 3*b and c*.

From Fig. 3*c*, the total density-thickness image was calculated, as shown in Fig. 3*d*. Although Fig. 3*d* is analogous to a conventional x-ray absorption image, where near black tones indicate the outside of the airway (since tissue and ASL have very similar densities and hence very similar absorption) and white tones indicate the airway lumen, the image of the thickness gradient (3*c*) is considerably more amenable to precise measurement of the ASL.

**Figure 4:**
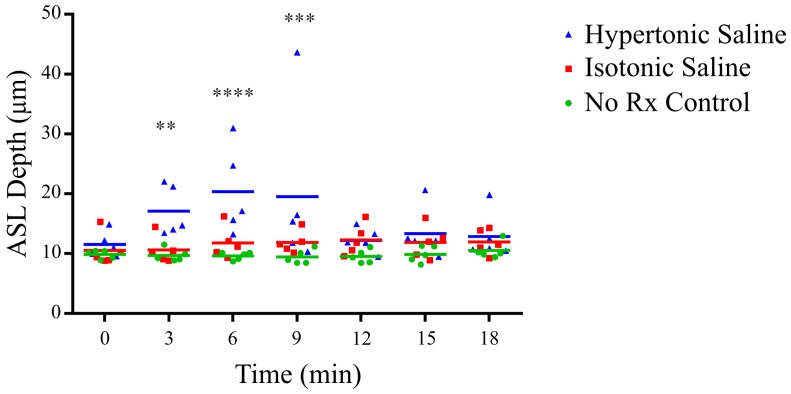
ASL depth as a function of time, in response to hypertonic saline (7%), isotonic saline (0.7%) or no treatment (No Rx – Control). A statistically significant increase in the ASL depth occurred with hypertonic saline treatment at 3 minutes (** p<0.01), 6 minutes (**** p<0.0001) and 9 minutes (*** p<0.001) compared to control.

Figure 4 shows that there was a statistically significant increase in ASL depth at 3, 6 and 9 minutes following hypertonic saline delivery, compared to the no-treatment group. In terms of absolute ASL depth, from a pre-treatment mean ASL depth of 10.6 µm for all groups, the hypertonic saline group reached an average maximum of 20.3 µm.

There were no significant differences in ASL depth observed between the normal saline and no-treatment groups.

The images in Fig. 3 also reveal the outer tissue interface, where the PBS bath surrounds the excised airway. To evaluate the effect of treatments on this surrounding tissue, measurements were also taken of the outer tracheal tissue thickness, averaging the distance between the ASL-tissue boundary and the tissue-bath boundary over the length of the airway between cartilage rings, in the same manner as the ASL depth measurements.

**Figure 5:**
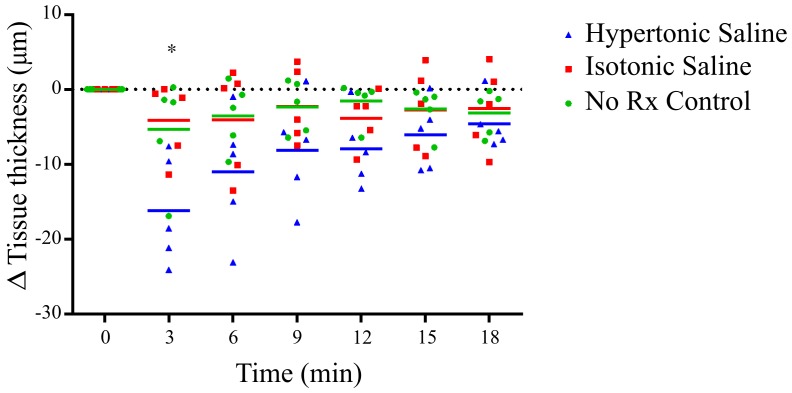
Change in tissue thickness (relative to initial thickness), as a function of time. A statistically significant difference (* p<0.05) in thickness was present for the hypertonic saline group at 3 minutes compared to control.

Figure 5 shows a statistically significant decrease in tissue thickness was present immediately after hypertonic saline treatment, with a trend showing a steady reduction in tissue thickness to the final 18 minute assessment point. There were no significant differences between the normal saline and no-treatment groups.

## Discussion

This study has demonstrated that our single-grid phase contrast x-ray imaging method [14] can visualise and quantify changes in ASL depth in freshly excised mouse tracheas. The effects of nebulised delivery of saline solutions of different tonicities on ASL depth were successfully measured.

Treatment with hypertonic saline produced a statistically significant increase in ASL depth, with the effect peaking 6–9 minutes after treatment before returning to baseline by 12 minutes. Previous studies with normal human epithelial cell cultures have found similar results [28], [8], with the transient increases in ASL depth also concluded by approximately 12 mins. In those epithelial cell culture studies, the initial ASL depth was approximately 10 µm (depending on mucus thickness) and increased to approximately 30 µm. When isotonic saline was delivered a slight but non-significant increase in total ASL depth was observed. This is consistent with deposition of the nebulised isotonic saline volume on the airway surface. We, therefore, attribute the increase in ASL depth in the presence of hypertonic saline to the osmotic imbalance driving water from and around epithelial cells onto the airway surface.

The evidence for this is two-fold. First, since those tracheas given hypertonic saline received the same volume of fluid as tracheas in the isotonic group, it is unlikely that the ASL increase came from simple deposition of a nebulised volume onto the airway surface alone. Second, changes in the volume of tissue/epithelial cells were observed, consistent with contribution of cellular fluid to the ASL. This was particularly noticeable immediately after treatment, as seen in Figure 5. This decrease in “tissue thickness” immediately following hypertonic saline delivery suggests that a volume of liquid left the epithelial cells across the more water permeable apical membrane in response to the aerosol-induced hypertonicity of the ASL. The decrease in tissue volume was approximately equal to the increase in ASL volume (around 10 µm), as would be expected. It is interesting to note that the minimum tissue volume is observed immediately following treatment, whereas the maximum ASL depth was on average not observed until two timepoints later. It should, however, be noted that we are observing only one view (or projection) covering around 15% of the total height of the trachea, hence liquid that is donated to the ASL by airway surface cells may move in a non-uniform way around the airway surface in this intervening time.

These results confirm that our grid imaging method can directly observe changes in ASL depth in *ex vivo* tissue samples. To our knowledge, this is the first time that such direct measurements of the ASL depth have been achieved in intact lengths of trachea. An important feature of the imaging set-up is that it is compatible with *in vivo* experiments without further modification. The primary remaining challenges for future *in vivo* investigations relate to the overlying anatomy which will tend to obscure the subtle tissue-surface liquid interface in imaging intact animals, and the expected x-ray dose, particularly for longitudinal or repeat imaging necessary for treatment efficacy studies. In the first case, we have some confidence from ongoing studies that simple steps such as removing the fur and gently stretching and tethering the skin of mice significantly ameliorates the effects of overlying structures [22]. Continued detector improvements are contributing to dose reductions via shorter exposure times. Other ongoing work at the SPring-8 synchrotron has shown that repeat imaging studies are possible, indicating that using these imaging methods to track treatments over longer periods is technically feasible. Further studies examining the effects of hypertonic saline, as well as other pharmaceuticals that modify airway surface function, can now be undertaken. Similarly, studies could examine differences after treatments in wildtype mice, and in the β-ENaC and CF-knockout mouse models.

## Conclusion

The development of new treatments for the life-shortening lung disease of cystic fibrosis is grounded in precise methods of airway health monitoring. We have shown that a new method of phase contrast x-ray imaging can directly measure the depth of airway surface liquid in a living *ex vivo* airway, with micron resolution. In the *ex vivo* airway, the increase in ASL depth in response to hypertonic saline was of the magnitude and duration expected from previous airway culture studies. This new imaging method can assist in progressing *in vivo* animal model imaging of the airways for the assessment of promising new treatments for cystic fibrosis.
