# Correction: Measuring Airway Surface Liquid Depth in Ex Vivo Mouse Airways by X-Ray Imaging for the Assessment of Cystic Fibrosis Airway Therapies

**DOI:** 10.1371/journal.pone.0097829

**Published:** 2014-05-15

**Authors:** The PLOS ONE Staff

There were errors in the Funding section. The correct funding statement is as follows: The authors acknowledge travel funding provided by the International Synchrotron Access Program (ISAP) managed by the Australian Synchrotron and funded by the Australian Government. This work is supported by the Australian Research Council, the National Health and Medical Research Council, Cure4CF Foundation Limited, and the NIH grant number NIH-P01- HL-110873 supported this publication. The funders had no role in study design, data collection and analysis, decision to publish, or preparation of the manuscript.
